# Branch Hospitals for Convalescents

**Published:** 1900-03-31

**Authors:** 


### BRANCH HOSPITALS FOR CONVALESCENTS.

A remark made by one of the speakers at a recent meeting of the Clinical Society raises some interesting questions concerning hospital management, and suggests a very important problem in regard to the after-care of discharged hospital patients. The remark to which we refer was to the effect that it was most difficult to give hospital patients the prolonged rest that was necessary for the proper treatment of acute rheumatism. There is no doubt about the difficulty, nor is it confined to cases of rheumatism; and, indeed, it is much to be feared that the desire to do as much good as possible with each hospital bed, and the presumed necessity of clearing out the patients as soon as they arrive at the stage when "active treatment" is no longer necessary, and nothing but rest is required, lead to many a patient going home before he is fit to return to the rough and tumble of ordinary life. It must be confessed that, in view of the crowds waiting for admission, and of the urgency of many of the cases which would be deprived of the special treatment which the hospitals alone can give them if partially convalescent patients were allowed to stay in the wards a day longer than is absolutely necessary there is much to be said for the position so generally taken by hospital managers that shortness of stay per inmate is a good criterion of crisp management. At the same time, it has also to be confessed that the principle of working a hospital at such high pressure involves hardship on many a patient. If we ask: How can we do the greatest amount of good with the limited number of beds at our disposal? the answer is plain enough. Turn out the chronics who only require rest, and fill up the beds with those who require active treatment, and for whose benefit we can make use of the highly-trained medical and surgical skill which we have so freely at our disposal. But if we come back to the individual rather than the average, and ask: What is the best for this particular patient? the answer will often be —keep him until he is strong, and able once again to return to work; consider his home circumstances, and if you find that he would have to return to poverty and to the sparse attentions which alone he could receive from a wife already hard put to it to keep herself and little ones until the bread winner gets to work again, think twice before you discharge your half-cured patient.

Turning to the tables contained in "Burdett's Hospitals and Charities," we find a heading giving the "proportion of in-patients to each bed occupied (average)," and under it we see how strikingly different is the average time of stay in the wards at different hospitals. Taking the hospitals with medical schools in London, we find that each bed served for 18 patients at the London, for 16 at St. Thomas's, and for only 13 at St. Batholomew's. Turning now to provincial hospitals with medical schools, we find in the same way each bed serving for 21 patients at the Birmingham General, for only 12 at the Liverpool Royal Infirmary, and at the Liverpool Southern for only 11. If now we take general hospitals in London without medical schools, we find that at the North-West London Hospital each bed only served for 12 patients in the year, while at the West London Hospital the corresponding number was 18, and at the Poplar Hospital 20. Among the provincial hospitals without medical schools the same thing is seen, the Royal Berkshire Hospital having only 9 patients per bed, while the Hull Royal Infirmary has 18, and the Cleveland Cottage Hospital 20. There may be special circumstances to explain special cases. Still, the wide differences in the number of patients dealt with per bed can, we think, in many cases only be accounted for on the hypothesis that the managers feel very differently on the important question of the treatment of the convalescent and partially convalescent patients whom they are constantly finding on their hands. We have to recognise that so far as charity is concerned—so far as the giving to the poor man what he cannot obtain elsewhere, and what to him may make all the difference between a long life and a short one—the offer of another month of bed may be a gift as precious to one as is the offer of some wonderful *tour de force* of scientific surgery to another. Yet a perfect hospital, built and managed on modern lines, is an expensive affair—too expensive to be used for those who merely require rest and a moderate amount of care during convalescence. We can hardly doubt, then, that one of the best, if not actually the best, ways of relieving the pressure upon our great general hospitals would be by a considerable extension of the branch-hospital system, so that each hospital would be able to send to its convalescent branch, which could be managed on comparatively cheap lines, such patients as are not yet fit for home and work, and yet do not require the expensive treatment provided at the central institution. In view of the constant hankering after bricks and mortar which all hospitals display, it is a matter of much interest to decide where all these bricks and all this mortar should be placed.

